# The effects of two different fatigue protocols on movement quality during anticipated and unanticipated change of directions in female soccer players

**DOI:** 10.1371/journal.pone.0302144

**Published:** 2024-05-22

**Authors:** Mohammad Alimoradi, Elham Hosseini, Mansour Sahebozamani, Thomas Dos’Santos, Shima Sheikhbahaie, Roya Bigtashkhani, Aryan Kabiri

**Affiliations:** 1 Department of Sports Injuries and Corrective Exercises, Faculty of Sports Sciences, Shahid Bahonar University, Kerman, Iran; 2 Musculoskeletal Science and Sports Medicine Research Centre, Department of Sport and Exercise Sciences, Manchester Metropolitan University, Manchester, United Kingdom; 3 Department of Health and Sport Medicine, Faculty of Physical Education and Sport Sciences, University of Tehran, Tehran, Iran; Università degli Studi di Milano: Universita degli Studi di Milano, ITALY

## Abstract

**Objective:**

This study compared neuromuscular control under two fatigue protocols during anticipated and unanticipated change of direction (COD) maneuvers and evaluated their effects on the risk of non-contact ACL injuries.

**Method:**

Forty-five female soccer players (mean age: 22.22 ± 2.24 years; mean height: 166.24 ± 3.33 cm; mean mass: 59.84 ± 5.03 kg) were divided into three groups: functional fatigue (Soccer specific fatigue ptotocol-SOFT90), non-functional fatigue (Bruce protocol), and control group. Before and after the implementation of neuromuscular control fatigue protocols were evaluated using the cutting motion assessment score tool (CMAS). Two-dimensional (2D) videos were recorded during anticipated and unanticipated COD trials for both dominant and non-dominant legs.

**Results:**

Significant time effects (p < 0.05) and group-time interactions (p < 0.05) were observed in both anticipated and unanticipated conditions for both dominant and non-dominant legs after the fatigue protocols. The functional fatigue group exhibited higher CMAS changes, indicating poorer movement quality following fatigue. Notably, the non-dominant leg displayed amplified deficits during unanticipated COD maneuvers following the functional fatigue protocol.

**Conclusions:**

Fatigue significantly impairs neuromuscular control, particularly in unanticipated COD situations, which increases the risk of non-contact ACL injuries. To mitigate this risk, coaches, trainers, and medical professionals should prioritize targeted training and injury prevention strategies, focusing on the non-dominant leg during unanticipated COD maneuvers.

## Introduction

In young athletes, knee injuries stand out as a prevalent concern, particularly in the form of knee ligament sprains [1]. Among these, the rupture of the anterior cruciate ligament (ACL) is recognized as the most severe consequence, often resulting in extended breaks from sports, increased vulnerability to re-injury, and a heightened risk of early onset osteoarthritis [2, 3]. Notably, female athletes face a greater susceptibility to knee and ACL injuries compared to their male counterparts [1]. Despite extensive efforts, ACL injuries remain common and these Injuries can cost a club up to £45 million in a single English Premier League season, or around 500 000 euros each player missing for a month [4]. Nearly 70% of ACL injuries occur through non-contact mechanisms, including during change of direction (COD) maneuvers [5, 6]. In soccer, non-contact mechanisms, especially frequent change of direction (COD) actions, are major contributors to ACL injuries [7]. The repetitive impact forces and mechanical loading during COD maneuvers can cause structural damage to the soft tissue around the knee joint [8, 9]. These forces and loads are further amplified by sub-optimal neuromuscular control and movement quality, which are modifiable factors affecting ACL loading and strain [5, 8].Therefore, optimizing neuromuscular control and movement quality during CODs is crucial for reducing non-contact ACL loading and injury risk in athletes [10]. It is common for athletes to be screened/profiled during a movement to evaluate their movement quality, joint kinetics, or kinematics in comparison to predetermined criteria or benchmarks. An injury-risk profile, which can be used to infer possible injury risk, can be created by investigating joint kinetics and kinematics and evaluating surrogates of injury risk. This can be accomplished by three-dimensional (3D) motion and ground reaction force analysis or high-speed two-dimensional (2D) cameras. In this method, movement is filmed (typically in the frontal and sagittal plane), and aberrant and "high-risk" movement patterns can be recognized through visual observations (e.g., visible knee valgus/lateral trunk flexion) [5].

Several internal and external risk factors, both modifiable and non-modifiable, contribute to increased ACL injury risk [11]. Fatigue, as a modifiable risk factor, can lead to altered neuromuscular control strategies and potentially increase ACL strain and injury risk [12, 13]. Soccer, being a high-intensity intermittent sport played over 90 minutes, often induces neuromuscular fatigue [14]. Acute or shot-term fatigue is a temporary condition of fatigue that is typically brought on by physical activity or mental strain. oeripheral fatigue is a type of acute fatigue resulting from muscle weakness. To improve a person’s performance, the main cause of fatigue must be identified and treated [15]. This fatigue can impact muscle activation, coordination, and subsequent joint coordination, resulting in poor movement quality and increased knee joint loads [16]. Such factors can impair the ability to perform accurate movements like COD, leading to reduced dynamic joint stability and heightened injury risk, especially during later stages of matches due to fatigue [17].

Numerous studies have investigated the effects of fatigue protocols on kinetic, kinematic, and electromyography changes during anticipated COD tasks in both injured and uninjured participants [18–20]. For instance, Hosseini et al. found that fatigue and unanticipated conditions during COD tasks increased the risk of knee injuries [21]. Fatigue can reduce sensory sensitivity and proprioceptive capacity in the lower limbs, affecting muscle balance and increasing the risk of knee injuries. Fatigue and unanticipated conditions further decrease neuromuscular control [19, 21]. Xia et al. studied the effects of two fatigue protocols on lower extremity impact forces and kinematics during drop landing tasks. Fatigue led to increased hip and knee flexion angles, resulting in a more flexed landing posture. The peak impact force and loading rate were similar before and after fatigue. Although the SV-FP (shuttle running + vertical jumping fatigue protocol) had a shorter exercise duration than R-FP (constant speed running fatigue protocol), the changes were similar [20]. Furthermore, the researchers reported that fatigue, repetition of COD manoeuvring, and unanticipated activities can change the kinematics of the lower limbs and trunk, exposing a person to non-contact ACL injury. However, the implementation of laboratory protocol results and comparison of results with the conditions experienced by participants of any sport on the real-world has been described as a challenge in the previous literature [22–26]. Many biomechanical fatigue studies lack ecological validity for sport-specific activities like COD in soccer, as they often focus on anticipated tasks and employ uncontrolled conditions [27, 28]. Previous researchers have also used non-specific fatigue induction methods that do not mimic the physical activity and movement patterns common in sports, such as the Bruce protocol on a treadmill, isokinetic strength measurement system, and PACER test [17, 21, 29]. Functional fatigue procedures aim to replicate the demands of a specific task or sport. They entail repeatedly performing the task or activity until fatigue occurs. This form of fatigue protocol is more similar to the demands of real-world activities than typical laboratory-based fatigue protocols, which frequently use isolated muscle contractions or basic tasks [30]. Because of this, outcomes from the generalization of functional protocols may differ from those obtained from methods used in laboratories.

This study aims to evaluate the effects of two fatigue protocols (non-functional and functional) on movement quality and ACL injury risk during both anticipated and unanticipated change of direction (COD) maneuvers in female soccer players. The hypothesis is that the functional fatigue protocol will result in poorer COD movement quality and increased ACL injury risk, particularly in unanticipated conditions. By examining these factors in both anticipated and unanticipated scenarios, we seek to enhance our understanding of the relationship between functional fatigue, knee biomechanics, and injury susceptibility in soccer players.

## Materials and methods

### Participants

The sample size was determined using G*Power software (version 3.1.9.4), which indicated that a minimum sample size of 33 was required to achieve 80% power, α = 0.05, and effect size of 0.5 for a repeated measures ANOVA. In total, 45 amateur Iranian female soccer players participating in the provincial league in Tehran city were recruited, considering potential drop-outs. The study was conducted during the in-season of soccer competitions, and none of the participants had prior experience in resistance training. Random.org website was used to randomly assign the participants into three groups: functional fatigue (FF), general fatigue (GF), and control (CO). The FF and GF groups performed the COD test before and after the fatigue protocols, while the CO group only performed the COD test. The test was conducted randomly in both anticipated and unanticipated conditions, and movement quality of both the dominant and non-dominant legs was measured. Inclusion criteria included no history of lower extremity surgery, no recent lower limb injury, at least 3 years of soccer experience, and regular training (3 sessions per week). Participants were familiarized with the experimental procedures and provided written consent before the study. The current study was approved by a research ethics committee in Iran (IR.UK.REC.1400.029), following the latest version of the Declaration of Helsinki.

### Procedure

**Fig 1:**
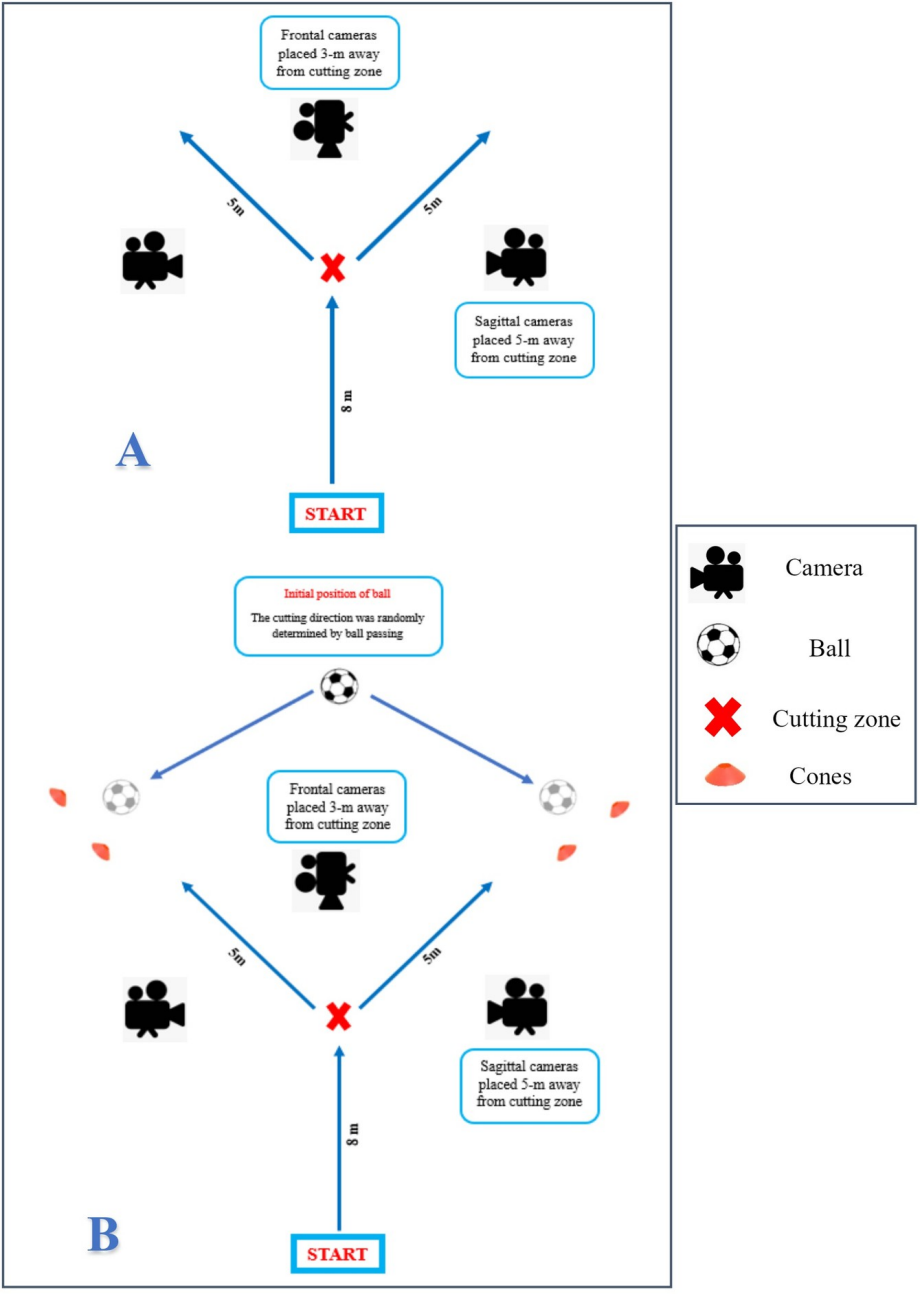
Schematic representation of performing COD by CMAS tool at 45°. (A) Predictable condition. (B) Unpredictable condition.

All participants were informed about the aims of the study and were familiarized with the data collection procedures. The all testing process was completed in four sessions between the first to the tenth of July, 2023. In this regard, the tests were evaluated over three separate sessions, with a 48-hour gap between each session. On the first day, participants completed a consent form and a personal information questionnaire. Their weight and height were also measured (participant characteristics data can be observed in Table 2). Next, the participants were randomly assigned to one of three groups: functional fatigue (FF), general fatigue (GF), or control (CO). On the second day, participants performed a warm-up program specifically designed for the lower extremities, including dynamic stretching exercises [31]. They also kicked a ball as far as possible to determine their leg dominance [32]. Kinematic variables were measured in two dimensions using Kinovea software (version 0.8.15 for Windows, Bordeaux, France). Three smartphones (iPhone 7, iOS 15.6) were mounted on tripods and positioned at a height of 0.6 m and a distance of 5 m in the sagittal plane, as well as 3 m in the frontal plane, to record videos of all COD trials for subsequent analysis. The videos were recorded at a frame rate of 120 frames per second (Fig 1). Before testing, each participant was shown the COD maneuver and allowed to perform three test trials to familiarize themselves with the actual tests. Participants were then given at least a 5-minute rest before the testing began. During the testing, participants were asked to perform the change of direction test (COD) with step over technique in both anticipated (towards the dominant leg) and unanticipated (to distinguish the movement path with ball pass) situations. Participants ran 8.8 metres at 60% of their calculated maximum speed and, upon reaching a distinctive designed setting, performed a COD maneuver at a marked angle (35-55-degree deviation from the main path) [33]. Participants also completed a 20-m sprint test to determine their maximum speed. The time taken to complete the task was measured using a timing gates system (Smartspeed, Fusion Sport, Australia). If a test subject’s running speed was less than 60% of their maximum running rate in a 20-meter recorded speed test, the COD test would be repeated. In an anticipated COD maneuver, each subject performed the test three times separately towards the dominant and non-dominant leg directions. For the unanticipated condition, the subject was instructed to respond by moving either to the dominant or non-dominant leg at a designated cutting zone based on the direction of a ball in motion. A researcher, who remained consistent throughout the experiment, kicked the ball towards two cones placed like a mini goal within a predefined area. The timing of the ball pass was crucial, designed to occur just as the participant reached a designated zone, one step before reaching the designated target area. Due to the inherent unpredictability of the sport being investigated, precise control over the ball’s speed was only achievable during video analysis. Additionally, any trials requiring repetition due to ball delivery inconsistencies were excluded from the final analysis [33, 34]. The test was performed three times separately for both the dominant and non-dominant leg directions in the unanticipated condition as well. Overall, participants performed the COD maneuver in both anticipated and unanticipated conditions in random order, resulting in a total of 12 trials. The third day involved different fatigue protocols for the GF and FF groups. The GF group underwent the Bruce treadmill test, while the FF group underwent the SAFT90 functional fatigue protocol [35, 36]. The CO group did not undergo any fatigue protocol. After completing the Bruce and SAFT90 protocols in the post-test, participants performed the COD maneuver in both the anticipated and unanticipated settings again. The Borg Rating of Perceived Exertion Scale (BRPES) was used to assess fatigue. Participants performed the fatigue protocol until they reached a score of 20 or maximal exertion. It is worth noting that in the BRPES, the level of fatigue is scored based on numbers ranging from 6 to 20 [14]. All participants underwent the same experimental tests as session two, but this time the SAFT90 functional fatigue protocol was adopted [37]. Following the testing sessions, the recorded videos were scored using the cutting movement assessment score (CMAS) [5]. The CMAS is a 9-item qualitative screening tool (Table 1) that evaluates hip, knee, foot, and trunk postures during side-step cutting [38]. In this study, two raters who were familiar with CMAS and had sufficient experience in assessing COD due to their participation in similar studies in the past independently evaluated each video. They assigned a quality score based on CMAS, and the agreement between the two raters in individual items and total scores was considered the final score for each participant [35, 37, 39].

**Table 1:** Cutting Movement Assessment Score tool (CMAS).

Recommended Camera		Variable	Observation	Score
**Penultimate contact**				
**Side/20–45°**	1.	Clear PFC braking strategy	Y/N	Y = 0/N = 1
**Final Contact**				
**Front/20–45°**	2.	Lateral leg plant distance (at initial contact)	Wide, Moderate, Narrow	Wide = 2, Moderate = 1, Narrow = 0
**Front/20–45°**	3.	Hip in an initial internally rotated position (at initial contact)	Y/N	Y = 1/N = 0
**Front/20–45°**	4.	Initial knee ‘valgus’ position (at initial contact)	Y/N	Y = 1/N = 0
**All 3**	5.	Foot not in neutral foot position (at initial contact)	Y/N	Y = 1/N = 0
**Front/20–45°**	6.	Frontal/transverse plane trunk position relative to intended direction of travel (at initial contact and during WA)	L/TR,U, M	L/TR = 2, U = 1, M = 0
**Side/20–45°**	7.	Trunk upright or leaning back throughout contact (at initial contact and during WA)	Y/N	Y = 1/N = 0
**Side/20–45°**	8.	Limited knee flexion during final contact ≤ 30° (during WA)	Y/N	Y = 1/N = 0
**Front/20–45°**	9.	Excessive knee ‘valgus’ motion (during WA)	Y/N	Y = 1/N = 0
**Low ≤ 3**	Moderate 4–6	High ≥ 7	Score	/11

Note: PFC: penultimate foot contact; WA: weight acceptance; L: lateral; TR: trunk rotation; U: upright; M: medial; Y: yes; N: no.

#### Bruce protocol

Participants performed the Bruce protocol on a treadmill (h/p/cosmos-mercury COS 10198 model, Germany). The protocol began with walking at 0.8 m/s and 0% gradient for 3 minutes. The speed and incline were then increased by 2% every 3 minutes until reaching 2.7 m/s and a 10% gradient in the final 3 minutes, or until participants reached volitional exhaustion. Ratings of perceived exertion (RPE) were obtained every three minutes using a 6–20 scale. The test was stopped if the participant’s heart rate exceeded 220 minus their age, or if they experienced angina, dizziness, nausea, or volitional termination [36].

#### SAFT^90^ protocol

The SAFT^90^ was designed based on time-motion analysis data from English Championship matches (Prozone®) to replicate the fatigue response of soccer match play. The course consists of a shuttle run over a 20m distance with four poles for participants to navigate using walking, jogging, side-stepping, and sprinting. Participants perform backward running or side-stepping around the first pole, followed by forward running through the course, navigating the middle three poles [35, 39].

Participants were verbally instructed using an audio CD to sustain movement intensity and perform activities during the SAFT^90^ course. The audio CD contained a 15-minute activity protocol that was randomly and intermittently repeated to match the duration of a typical game. The activities included standing, walking, jogging, running, and sprinting, with reported speeds based on the average requirements for each activity. The 90-minute protocol was divided into two 45-minute sections, each consisting of three 15-minute periods. Participants completed three 15-minute periods in the first half, followed by a 15-minute rest before completing the second half in three 15-minute periods [35]. Furthermore, during the protocol, all participants were strongly encouraged with verbal encouragement to maintain their effort.

#### Statistical analysis

SPSS 26.0 software (IBM SPSS, Armonk, NY, USA) was used to perform all statistical analyses. The normal distribution was evaluated using the Shapiro-Wilk test. The one-way ANOVA test was used to compare demographic characteristics and pre-fatigue results in the two legs seperatly among the three groups. All variables were subjected to a mixed-design analysis of variance (ANOVA) with the time effect (pre-test and post-test) as the within-subject factor and the group effect (GF, FF, and CO) as the between-subject factor, including the group-time interaction (Three-way mixed ANOVA). When the group effect or interaction effect was significant, a post-hoc Bonferroni test was conducted for pairwise comparisons. The effect size (ES) was assessed using partial eta squared and classified as small (pη2 = 0.01), medium (pη2 = 0.06), or large (pη2 = 0.14).^33^ An alpha level of 0.05 was considered for all comparisons. Furthermore, the inter-rater reliability and intra-rater reliability were assessed using ICC values. ICC results below 0.50 are considered poor, values ranging from 0.50 to 0.75 are regarded as moderate, values ranging from 0.75 to 0.90 are considered good, and values exceeding 0.90 are classified as excellent [40].

## Results

The demographic data of the participants are available in Table 2. The one-way ANOVA test results showed that there was no significant difference between groups in the participants’ characteristics. The Shapiro-Wilk test indicated that all variables had a normal distribution (*p* > 0.05). The ICC obtained for inter-rater reliability was 0.88 (95% CI 0.75–0.93), indicating good to excellent agreement between the raters. Similarly, the ICC obtained for intra-rater reliability was 0.79 (95% CI 0.74–0.84), indicating good agreement between the raters.

**Table 2:** Demographic information of participants.

variables	GF	FF	CO	*P*
**Age (y)**	22.66 (2.46)	21.46 (1.68)	22.53 (2.44)	.28
**Mass (kg)**	60.73 (4.60)	61.06 (4.96)	57.73 (5.14)	.13
**Height (cm)**	167.26 (2.86)	165.53 (9.83)	165.93 (3.21)	.33
**Experience (y)**	4.00 (1.00)	4.40 (1.12)	4.86 (1.84)	.23
**BMI (kg/m2)**	21.73 (1.93)	22.30 (1.91)	20.96 (1.79)	.16
**20-m sprint test**	4.43 (0.65)	4.39 (0.53)	4.54 (0.70)	.79

Note: GF = general fatigue; FF = functional fatigue; CO = control

Significant level set as *P* < 0.05.

No significant differences were found in pre-fatigue measurements between groups in both the dominant and non-dominant legs for both anticipated and unanticipated conditions (*p* > 0.05). However, following the fatigue protocols (GF and FF) in the anticipated condition, significant time effects (*p* < 0.05) and group-time interactions (*p* < 0.05) were observed, with large effect sizes for both legs (see Table 3). The group effect was not significant for the dominant and non-dominant legs, but the FF group had higher mean scores, indicating more errors during CMAS after fatigue.

**Table 3:** The records (Mean (SD) of studied groups (GF, FF, CO) in pre–fatigue and post–fatigue, percentage of change (Δ) (values are (post-test-pre-test/pre-test) ×100)).

Groups	Anticipated												Unanticipated											
	Dominant						Non-dominant						Dominant						Non-dominant					
	Pre-fatigue	Post-fatigue	Δ%	Time effect	Group effect	Group × Time	Pre-fatigue	Post-fatigue	Δ%	Time effect	Group effect	Group × Time	Pre-fatigue	Post-fatigue	Δ%	Time effect	Group effect	Group × Time	Pre-fatigue	Post-fatigue	Δ%	Time effect	Group effect	Group × Time
**GF**	2.93(1.57)	3.40(1.45)	16.04	F = 13.78	F = 0.30	F = 3.60	4.00(1.19)	4.53(1.06)	13.25	F = 31.97	F = 0.24	F = 20.48	4.73(1.03)	5.00(1.06)	5.70	F = 20.06	F = 0.82	F = 9.71	5.06(1.03)	6.20(1.26)	22.52	F = 108.33	F = 1.93	F = 38.48
**FF**	2.86(1.50)	3.46(1.30)	20.97	*P* = .001	*P* = .78	*P* = .03	3.93(1.48)	5.33(1.39)	35.62	*P* = .001	*P* = .78	*P* = .001	3.86(1.12)	5.13(1.12)	32.90	*P* = .001	*P* = .44	*P* = .001	4.60(1.24)	7.66(1.29)	66.52	*P* = .001	*P* = .15	*P* = .001
**CO**	3.53(1.59)	3.53(1.64)	0	ES = 0.24	ES = 0.01	ES = 0.14	4.53(1.76)	4.33(1.79)	- 4.41	ES = 0.43	ES = 0.01	ES = 0.49	4.93(1.09)	5.00(1.25)	1.41	ES = 0.32	ES = 0.03	ES = 0.31	5.26(1.16)	5.40(1.29)	2.66	ES = 0.72	ES = 0.05	ES = 0.64

Note: GF = general fatigue; FF = functional fatigue; CO = control; ES = effect size

Significant level set as *P* < 0.05.

**Fig 2:**
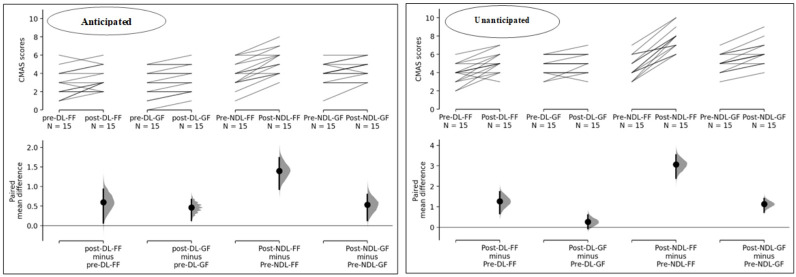
Comparison CMAS scores of FF and GF groups in anticipated and unanticipated conditions. FF = functional fatigue; GF = general fatigue; DL = dominant limb; NDL = non-dominant limb.

**Fig 3:**
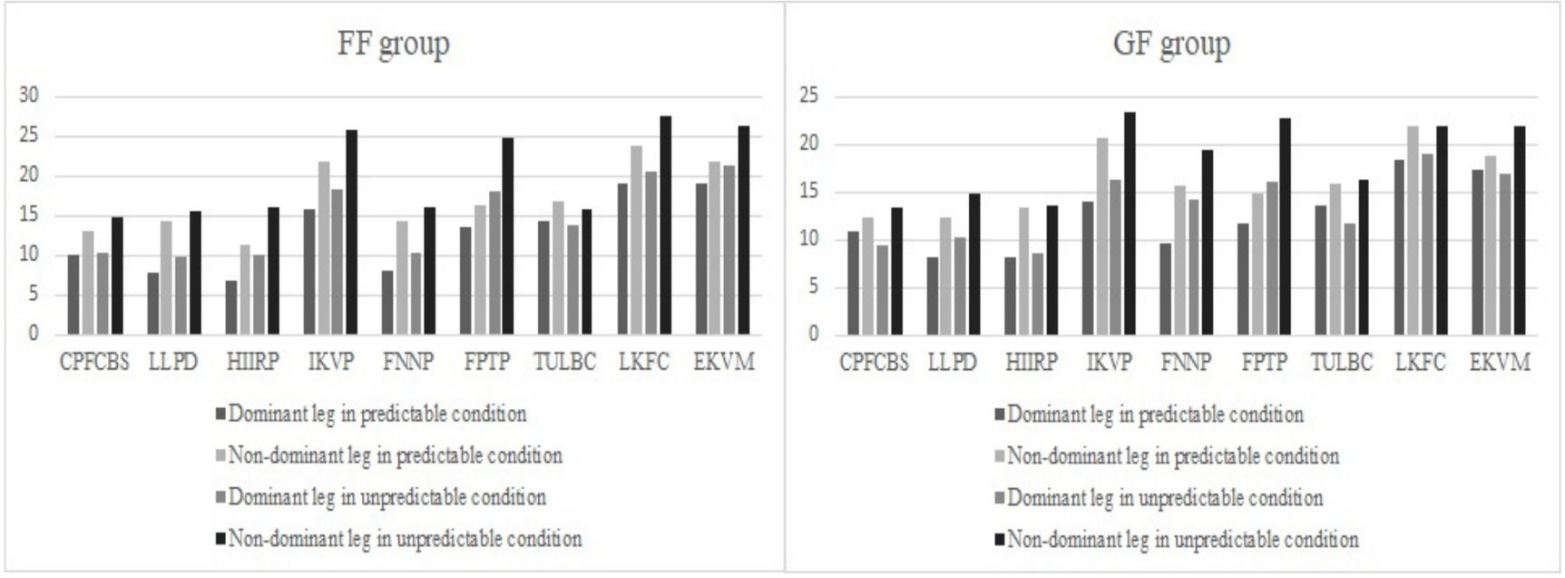
Represents changes in the items of CMAS following fatigue protocols in the GF and FF groups. FF = functional fatigue; GF = general fatigue; CPFCBS = Clear PFC braking strategy; LLPD = Lateral leg plant distance; HIIRP = Hip in an initial internally rotated position; IKVP = Initial knee ‘valgus’ position; FNNP = Foot not in neutral foot position; FPTP = Frontal/transverse plane trunk position relative to intended direction of travel; TULBC = Trunk upright or leaning back throughout contact; LKFC = Limited knee flexion during final contact; EKVM = Excessive knee ‘valgus’ motion.

Significant differences with large effect sizes were also observed in both the dominant and non-dominant legs, specifically in the time effect and group-time interaction, under unanticipated conditions following the fatigue protocols. Although the group effect in both legs did not reach statistical significance, it is important to note that no observable differences among the groups were found, accompanied by small effect sizes. Comparing the changes in CMAS items after the fatigue protocols in the GF and FF groups (as illustrated in Figs 2 and 3), it was demonstrated that in the anticipated condition, the GF group had the most changes in 8 items (dominant leg: 18.32%, non-dominant leg: 21.84%) and 9 items (dominant leg: 17.24%, non-dominant leg: 20.62%). The FF group showed the most changes in 8 items (dominant leg: 19.08%, non-dominant leg: 23.69%) and 9 items (dominant leg: 19%, non-dominant leg: 21.83%) in the anticipated condition. In the unanticipated condition, the GF group had the most changes in 8 items (19%) and 9 items (17%) for the dominant leg, and 4 items (23.42%) and 6 items (22.84%) for the non-dominant leg. The FF group demonstrated the most changes in the unanticipated condition in 8 items (dominant leg: 20.64%, non-dominant leg: 21.33%) and 9 items (dominant leg: 27.54%, non-dominant leg: 26.28%).

## Discussion

This study examined the effects of fatigue protocols on cutting movement quality in the dominant and non-dominant lower limbs under anticipated and unanticipated conditions. The findings showed that fatigue led to decreased knee flexion and increased knee valgus, particularly in the non-dominant leg across all conditions. Additionally, the FF group exhibited greater changes in knee flexion and knee valgus compared to the GF group. The results of our study are in line with previous studies. For instance, Needham et al. observed that female football players exhibited a higher CMAS when performing an unanticipated change of direction (COD) task as opposed to an anticipated COD task. Consequently, these athletes are more susceptible to exhibiting movement patterns that are considered ’high-risk’, thereby increasing their risk of injury [33]. The response to a dynamic object, such as a movable ball, could potentially influence the observed outcomes. Therefore, it is recommended that additional investigation be conducted on unforeseen COD tasks to ascertain the underlying causes for these disparities and to assess the influence of anticipation on task performance [33]. Moreover, Nijmeijer et al. found that providing feedback utilizing the Cutting Movement Assessment Score (CMAS) led to enhanced learning and greater advancements in movement execution compared to the control group in the anticipated condition. Consequently, the validity of the CMAS scoring tool in the context of COD maneuvers has been acknowledged through various studies [41]. Additionally, Hosseini et al. found that combining a fatigue protocol with an unanticipated condition resulted in a smaller knee flexion angle and increased knee valgus angle in the non-dominant leg compared to the dominant leg during COD [21]. Similarly, Schmidt et al. demonstrated that in unanticipated conditions, fatigue caused the knee of the non-dominant leg in female handball players to be more extended during jumping and cutting movements [42]. Previous research suggests that the combination of fatigue and decision-making hampers proprioceptive receptor function, increasing the risk of injury. Yom et al. found that unanticipated function exhibited unsafe performance with notable increases in kinematic and kinetic risk factors in the lower extremities [43]. Borotikar et al. found that fatigue can increase cognitive errors, impaired decision-making and concentration in athletes and exposed them at risk of injury [44]. In addition, a systematic review found that fatigue affects several cognitive contexts. They also reported that fatigue could affect a decrease in the ability to process Environmental Information, predict events and respond appropriately [45]. furthermore Bertozzi et al. found that fatigue reduced unforeseen landing mechanics, as neuromuscular coordination and the ability to predict the moment and place of contact on the ground reduced limb posture and stress absorption, and placed the athlete in the risk pattern of non-contact injury factors ACL [46]. Our study, along with previous research, suggests that the dominant leg may have superior neuromuscular control compared to the non-dominant leg. However, the rate of change in these parameters varied, likely due to different testing conditions and participants [43].

In the present study, our results showed that the functional fatigue protocol (SAFT90) promoted greater changes in knee mechanical factors than general fatigue (Bruce protocol) when combined with an unanticipated condition. Previous literature suggests that less knee flexion and greater knee valgus are key factors that increase ACL strain [47]. These results are consistent with Olsen et al., who indicated that during change of direction (COD) and forward movement, the body accelerates in the opposite direction, reducing the knee flexion of the non-dominant leg while increasing knee valgus. These findings suggest that neuromuscular control in the dominant leg is superior [48]. Despite this evidence, however, studies by Pollard et al. and Brown et al. demonstrated that fatigue or limb dominance did not result in any significant changes in kinematic characteristics [49, 50]. Possible causes of this discrepancy include variations in the testing setting, fatigue methods, and subject conditions.

As the functional fatigue protocol includes a variety of movement patterns such as running, deceleration, and change of direction (CODs) to replicate actual situations during matches or training for soccer players, this multi-movement protocol may impair neuromuscular control and increase ACL injury risk to a greater extent than the Bruce protocol. Quammen et al. studied the effects of two fatigue protocols on lower extremity biomechanics of female NCAA Division I soccer players during an unanticipated running-stop jump task [51]. They found a decrease in knee and hip flexion after the protocols, leading to a more erect or extended position [51]. This increased anterior shear force on the tibia and decreased joint angles may disadvantage hamstring muscles, increasing ACL loading [51]. In their study, the FAST-FP (functional agility short-term fatigue protocol) induced changes in frontal-plane hip and knee biomechanics compared to the SLO-FP (slow linear oxidative fatigue protocol). Hip abduction at peak knee flexion was greater during the FAST-FP, possibly due to greater fatigue in the hip musculature. Additionally, increased hip abduction angle during landing and knee valgus and abduction moments were observed after FAST-FP fatigue. The researchers stated that the FAST-FP or multidirectional functional protocol recruited various muscle groups, while the SLO-FP primarily affected flexors and extensors [51]. Schmidt et al. also discovered that functional fatigue affected the initial knee flexion angle of the non-dominant limb in all tasks [42]. Moreover, significant changes were observed in the initial knee flexion angle of the dominant limb, the initial and maximum hip flexion angle, and the maximum knee flexion angle of the non-dominant leg during the cutting task [42]. In general, their findings revealed that fatigued players are more susceptible to high-risk movement patterns, particularly in their non-dominant limb. Furthermore, Arsalan et al. found that soccer match-induced neuromuscular fatigue affects landing biomechanical parameters, indicating a sensory rather than motor cause [14]. The study emphasizes the importance of considering the impact of fatigue on impact biomechanics, neuromuscular performance, and movement quality to prevent injuries. Training programs should address soccer match-induced fatigue to mitigate its effects on landing errors [14]. However, ACL injury risk factors are multifactorial, involving biomechanical and neuromuscular deficits. Therefore, injury prevention programs are recommended to address these factors in both fatigued and non-fatigued conditions. Implementing prevention programs in various situations allows athletes to train safely and improve identified neuromechanical risk factors [14].

This study had limitations to consider. Firstly, qualitative evaluations were used for cutting tasks without direct, objective quantitative data on kinematics and kinetics. For instance, tibial rotation, known to be a potential risk factor for non-contact ACL injuries during change of direction (COD) maneuvers, lacked specific measurement in this study [52]. Thus, future research should examine the effect of the fatigue protocols on knee kinematics and kinetics using three-dimensional motion analysis and ground reaction force (GRF) analysis. That said, the Cutting Movement Assessment Score (CMAS) has been validated against 3D motion analysis and is strongly associated with external peak knee abduction moments (KAMs) [53]. Secondly, we lacked The literature on longitudinal risk factors for anterior cruciate ligament (ACL) injuries in soccer players. Finally, the study’s small sample size was limited to Iranian soccer players. Future research should include larger sample sizes and encompass players with higher training status to enhance understanding of fatigue’s impact on movement quality.

## Conclusions

The study revealed that fatigue protocols worsened neuromuscular control and amplified deficits during unanticipated CODs in female soccer players, increasing the risk of non-contact ACL injuries. Specifically, cutting movement quality deficits in the non-dominant leg were heightened after the functional fatigue protocol, suggesting an elevated injury risk. These findings emphasize the need to enhance athletes’ fatigue resilience and reinforce movement quality during directional changes.

## Supporting information

S1 Checklist Human participants research checklist. (DOCX)
